# The Hungarian adaptation of the work-related Burnout Assessment Tool (BAT)

**DOI:** 10.1186/s40359-026-04711-2

**Published:** 2026-05-07

**Authors:** Georgina Csordás, László Dorner, Boglárka Faragó, Kitti Mária Kiss, Dolli Mester

**Affiliations:** 1 Department of Developmental and Educational Psychology, Eszterházy Károly Catholic University, Eger, Hungary; 2 Department of School Psychology, Eszterházy Károly Catholic University, Eger, Hungary; 3 Department of Ergonomics and Psychology, Faculty of Economic and Social Sciences, Budapest University of Technology and Economics, Budapest, Hungary

**Keywords:** Burnout Assessment Tool, Burnout, Factor analysis, Work-related burnout

## Abstract

Burnout has been widely studied due to its negative impact on employee well-being and performance. The Burnout Assessment Tool (BAT) was developed to provide a more comprehensive assessment of burnout, capturing both core and secondary symptoms within a unified framework. In this model, the core symptoms of burnout are exhaustion, mental distance, and impaired emotional and cognitive control, while secondary symptoms include psychological stress and psychosomatic complaints.

The present study examines the psychometric properties of the Hungarian workplace version of the Burnout Assessment Tool, which was published by the original authors as well, in a Hungarian sample (*N* = 1398, 66% female, mean age = 36.82 years, 30.76% with secondary education, 34.23% with a university degree). For validity testing, the Satisfaction with Life Scale, the Satisfaction with Work Scale, the Basic Psychological Needs at Work Scale, and the Work and Meaning Inventory were also administered. Confirmatory factor analysis supported the original factor structure formulated by the original authors (fit indices: χ² = 2084.55, *p* < .001; SRMR = 0.05; RMSEA = 0.06 (CI = 0.06–0.07), CFI = 0.90; TLI = 0.89), and the instrument demonstrated adequate reliability and validity. Additional analyses were conducted across age groups, university student status, and occupational groups. Overall, the findings indicate that the Hungarian version of the BAT is a reliable and valid instrument for assessing burnout in the workplace.

## Background

In contemporary society, burnout is one of the most prevalent psychosocial and occupational risk factors. Initially, burnout was conceptualized as a syndrome primarily affecting individuals whose professions involve working with people; however, it has become increasingly clear that the problem may arise across a wide range of occupational fields. Workplace conditions such as high-performance expectations or intense workloads place a substantial emotional burden on employees. In addition, with the widespread use of modern technological tools, employees are often accessible beyond regular working hours, which may further intensify psychological strain [1].

Measurement instruments designed to assess burnout can be divided into two categories: general and occupation-specific tools. The latter are tailored to assess burnout within specific professional groups (e.g., psychologists), whereas the former are not restricted to any one occupational field but evaluate burnout more broadly (the key differences among these instruments lie in the theoretical models they use to conceptualize burnout) [1].

### The “gold standard” of burnout measurement – the maslach burnout inventory

The most widely used instrument for assessing burnout is the Maslach Burnout Inventory (MBI) [2], which defines burnout as a syndrome characterized by emotional exhaustion and cynicism, occurring most frequently among individuals who engage in intensive interactions with others in their work (e.g., teachers, healthcare professionals). The first version of the MBI was specifically developed for measuring burnout in these occupational groups. According to the authors, the leading symptom of burnout is the overwhelming feeling of emotional exhaustion, while other symptoms include a negative, cynical attitude toward others and a decline in professional performance [2]. Subsequently, a general version of the MBI was developed, which could be applied beyond the aforementioned occupational groups [3]. This questionnaire defines burnout in broader terms: a state of exhaustion in which individuals display cynicism toward their profession and experience doubts about their own performance. In this questionnaire, exhaustion is no longer attributed to interactions with others; rather, cynicism refers to the work itself rather than to interpersonal relations at work, and the issues addressing professional efficacy are framed in a much broader sense, extending beyond social interactions [4].

The popularity of the Maslach Burnout Inventory is illustrated by the fact that, in 2015, 88% of scientific publications on burnout employed this instrument to assess the construct [5].

A meta-analysis reviewing studies on the psychometric validity of the general version of the MBI (covering 17 studies published between 1996 and 2022) highlighted that the measurement characteristics of the general version of the MBI raise certain concerns. In studies using the original 16-item version, the three-dimensional model of burnout (emotional exhaustion, cynicism, reduced performance) was supported; however, in 51% of these studies, this was only achieved after certain modifications (e.g., deletion of certain items). As a result, the structural validity of the general MBI remains questionable. Internal consistency was moderate (internal consistency above 0.70 was found for all dimensions; only in the case of exhaustion was internal consistency above 0.80 more likely to occur). According to the MBI manual, a unified score cannot be calculated from the questionnaire; however, the authors of the meta-analysis emphasize the need for a unified score, given that burnout is a syndrome comprising interconnected symptoms. The criterion validity of the cynicism and personal accomplishment scales also raises concerns, as although the factors of the general MBI correlated with other burnout measures, these instruments primarily targeted the measurement of exhaustion [6].

Schaufeli and colleagues [7] identified three fundamental problems with the Maslach Burnout Inventory: one conceptual, and two related to psychometric and technical issues.

The conceptual problem with the Maslach Burnout Inventory lies in the process of constructing the questionnaire. The question sheet was constructed using an inductive, bottom-up approach: researchers interviewed individuals from occupational groups considered to be at high risk of burnout (though these individuals were not yet displaying severe symptoms of burnout, but were assumed to be vulnerable). Based on these interviews, 47 items were created to assess burnout and administered to 600 participants working in the human services sector. Factor analysis revealed ten factors, of which four were central—the three already described, plus lack of involvement as a fourth. The number of items was then reduced to 25, and this version was tested with 400 participants. In total, 1,000 individuals were surveyed; however, this was not a representative sample of American human service workers, thus, no normal distributions are related that can be used in the test. Later studies also revealed that burnout is associated with reduced cognitive performance, yet this dimension is absent from the Maslach Burnout Inventory. Finally, it remains unclear whether reduced performance is a cause or a consequence of burnout—whether it should be considered a core symptom. In the former case, mental exhaustion arises when job performance declines; in the latter, reduced performance is the result of exhaustion [7].

The technical problem with the Maslach Burnout Inventory is that some items are formulated in an extremely strong way, which undermines the reliability of the questionnaire. Another issue is that the spacing between response options is inconsistent. Moreover, the questions relating to personal accomplishment are positively worded. As a result, low scores on this dimension indicate burnout, whereas for the other dimensions, higher scores indicate greater burnout. In other words, this dimension behaves differently from the other two burnout dimensions (emotional exhaustion and cynicism), leading to substantially lower correlations with them than they show with each other, which may cause statistical distortions [7].

Finally, a further practical problem with the Maslach Burnout Inventory is that it does not yield a single unified burnout score; the three scales of the questionnaire must be interpreted as separate dimensions. This makes the questionnaire unsuitable as a diagnostic tool for identifying individuals with severe burnout symptoms. Furthermore, no clinically validated cutoff values exist that would allow for the identification of individuals suffering from severe burnout, which limits the instrument’s applicability in practice [4].

For a long time, the Maslach Burnout Inventory was considered the most important instrument for assessing burnout. As a result, the definition of burnout became intertwined with what this questionnaire measured. However, this significantly restricts both the applicability of findings and the potential for achieving a deeper understanding of burnout [4].

### The development of the Burnout Assessment Tool (BAT)

Although several questionnaires have been created to measure burnout, none fully meet al.l the requirements outlined above in measuring burnout. To address this gap, a new instrument was developed by Schaufeli and De Witte that is suitable for assessing burnout both at the group and individual level: the Burnout Assessment Tool (BAT) [7].

The design of the BAT followed the following guiding principles. First, it combined deductive and inductive approaches while creating the questionnaire: the construction of the questionnaire was not only based on interviews and reviews of earlier burnout measures but also on theoretical considerations, particularly the literature describing two central characteristics of burnout: an inability to exert effort and a lack of willpower. As a second principle, up-to-date research findings were incorporated while creating the questionnaire items, recognizing reduced cognitive functioning (e.g., poor attention and concentration, limited working memory performance) as part of burnout, while reduced professional performance was treated as a consequence of effort depletion or lack of willpower. As a third principle, special care was taken in item formulation, such as avoiding reverse-coded items and using five-point Likert scales for response options. The fourth principle is that the new questionnaire should also be suitable for diagnostic use, i.e., the person administering the questionnaire should also receive a burnout total score, the dimensions of burnout should be interpretable independently, and clinically usable thresholds should also be available for diagnostic work. Finally, the fifth principle was to create a context-independent instrument, not tied to any particular occupational group [4].

The development of the BAT proceeded in four steps. First, in-depth interviews were conducted with professionals regularly encountering burnout in their work (e.g., physicians, psychologists). During these interviews, symptoms of burnout were collected and categorized. A total of 260 symptoms were identified and grouped into 19 categories, from which seven dimensions emerged: exhaustion, mental distance, impaired emotional control, impaired cognitive control, depressive mood, psychological distress, and psychosomatic complaints. These were further divided into primary symptoms (exhaustion, mental distance, impaired emotional control, impaired cognitive control) and secondary symptoms (psychological distress, psychosomatic complaints, depressive mood). Secondary symptoms may also appear in other physical or psychological disorders. A reconceptualization of burnout was also undertaken [4], which will be discussed later.

In the second step, questionnaire items were generated. The authors first reviewed the already existing burnout measures, a total of 13 questionnaires, to identify dimensions consistently appearing as symptoms of burnout (such as mental exhaustion, which was present in all, and mental distance). Each author then created five items for each of six dimensions, yielding a pool of 90 items. These were discussed and refined into a 33-item version of the BAT, covering four core dimensions and two secondary dimensions. The depressive mood dimension was not included as a subscale, and its assessment is instead recommended using a dedicated depression measure; the six-item depression subscale of the 4-DSQ [7].

The third step was to test the psychometric properties, validity, and reliability of the BAT. Finally, the fourth step established norms and cutoff scores with potential diagnostic relevance, based on individuals who were actually experiencing burnout at the time of assessment [4].

### A new conceptualization of burnout

As noted earlier, the first step in developing the new questionnaire was to reconceptualize burnout. The definition was formulated as follows: *“a work-related state of exhaustion that occurs among employees*,* which is characterized by extreme tiredness*,* reduced ability to regulate cognitive and emotional processes*,* and mental distance. These four core dimensions of burnout are accompanied by depressed mood as well as by non-specific psychological and psychosomatic complaints”* (pp. 4) [7].

The core symptoms of burnout include: (1) exhaustion, (2) mental distance, (3) impaired emotional control, and (4) cognitive control. (1) Exhaustion is a state characterized by severe lack of energy, which can affect the individual both mentally and physically. (2) Mental distance refers to psychological withdrawal from work, reluctance, or aversion toward work. (3) A person with impaired emotional control is overwhelmed by emotions and displays intense emotional reactions. (4) Impaired cognitive control involves memory problems, deficits in attention and concentration, and reduced cognitive performance. The secondary symptoms are the following: (1) psychological distress, which includes non-physical symptoms resulting from a psychological problem (e.g., sleep problems, worry, anxiety). (2) Psychosomatic symptoms, which are physical symptoms not caused by physical factors or problems but by psychological ones (e.g., digestive problems, chest pain not attributable to a physical cause). Finally, (3) depressed mood, the inability to experience pleasure, and a dark and gloomy mood state. According to the model, during burnout, extreme exhaustion leads to impaired emotional and cognitive control, which results in mental distance. The authors state that this distance appears as a kind of defense mechanism against burnout, but it is not truly adaptive, since it shows stress-increasing effects on several levels instead of reducing it (e.g., it diminishes motivation and performance and may also provoke negative reactions from colleagues). In this way, mental distance further fuels the individual’s exhaustion, impaired cognitive and emotional control, the experience of which reinforces the secondary symptoms of burnout [4].

### Variants of the BAT and their practical applicability

The authors of the original Burnout Assessment Tool also developed a general version, in which the concept of work is viewed from a broader psychological perspective. In this framework, *work* is defined as structured, goal-oriented activities that are obligatory for the individual, and thus not limited to paid employment. This makes it possible to study the phenomenon of burnout in individuals who do not receive payment for their work (e.g., students), or in those who are not currently employed because they are absent due to long-term illness (including burnout itself). The questionnaire consists of 33 items in total (23 addressing the core symptoms and 10 addressing the secondary symptoms). It does not include a depression subscale, since other brief, validated questionnaires are available for that purpose. Responses are given on a five-point Likert scale [4].

For the sake of efficiency, it is useful to have accurate and reliable yet brief measurement tools. This way, the information obtained from participants can be maximized while minimizing the time required for assessment. To this end, a short version of the BAT was created. Item selection was based on Rasch analysis and content analysis, and the original questionnaire was reduced to 12 items. This short version contains four subscales: exhaustion, mental distance, impaired emotional control, and impaired cognitive control (with three items per scale) [8]. Subsequently, an ultra-short version of the questionnaire was also developed, the four-item BAT4. Its construct validity was also analyzed using Rasch analysis across eight countries (the Netherlands, *N* = 1500; Belgium *N* = 1500; Austria *N* = 1054; Czech Republic *N* = 964; Finland *N* = 733; Germany *N* = 1073; Ireland *N* = 426; Japan *N* = 1028). The BAT4 proved to be a valid measurement instrument both collectively across all eight countries and individually within each country [9].

The questionnaire can be applied both for individual and group assessments. In organizational settings, group assessments help to estimate the prevalence of burnout, that is, to determine how many employees are at risk of burnout and how many are currently suffering from it. It can also be applied to employees as well as those on long-term leave. Monitoring burnout in this latter group may be useful in optimizing return-to-work processes, particularly if the leave is due to burnout itself. The questionnaire also provides a unified score, but the four subscale scores (impaired emotional control, impaired cognitive control, mental distance, and exhaustion) can be meaningfully interpreted independently, too [4].

Based on representative samples from Flanders (*N* = 1500) and the Netherlands (*N* = 1500), the authors established cut-off values, which can be highly useful in diagnostic work within these populations. The questionnaire shows strong discriminatory power, meaning that it can effectively distinguish between individuals with and without severe burnout symptoms [4]. In a subsequent study, data were collected in three countries (the Netherlands (*N* = 1370), Belgium (*N* = 1403), and Finland (*N* = 1350)) from representative samples of healthy individuals as well as from those diagnosed with burnout. Both the longer and the shorter versions of the BAT were used. Using ROC analysis, clinically validated cut-off values were determined for diagnosing burnout. Based on a *traffic light model*, individuals were classified into three groups: those suffering from severe burnout were labeled red, those at risk of burnout were labeled orange, and those without signs of burnout were labeled green. The identified cut-off values were highly similar across the three countries. General cut-off values applicable to all three countries were also defined, and these performed comparably well to the country-specific values, apart from the *mental distance* scale. This scale was somewhat less effective in distinguishing between individuals with and without burnout, leading the authors to caution that its cut-off values should be interpreted carefully in future research [10].

Both the BAT23 and the BAT12 proved to be suitable instruments. The short version is particularly advantageous for assessing burnout levels in groups of employees. For individual assessment, and especially for identifying who is at risk of burnout, the BAT23 is more appropriate, as it yields more precise results—given its larger number of items and higher discriminatory power, it can more accurately differentiate between affected and unaffected cases [4]. The BAT4, in turn, may be especially useful when the aim is to conduct a rapid, group-level screening within a specific organization or population, in order to identify individuals at risk of burnout [9]. Currently, the BAT has already been validated in a number of countries, such as: Portugal [11], Italy [12–15], Croatia [16], Greece [17], Lithuania [18], Norway [19], Romania [20], Ecuador [21], Brazil [11], Japan [22] and South Africa [23]. It should be noted that there is also a short version of the BAT, the BAT-12, whose discriminative power and psychometric properties are similar to those of the full version [4].

### Psychometric properties of the BAT

The psychometric properties of the Burnout Assessment Tool (BAT) were investigated in representative samples of Flemish (*N* = 1500) and Dutch (*N* = 1500) respondents. The psychometric analysis confirmed the hypothesized model, namely that burnout is a syndrome with four core symptoms. The results showed that while the four central dimensions were distinct, they were also closely related. This means that not only can the scores of the core dimensions be interpreted separately, but they can also be combined into a single unified score of burnout [4]. The construct validity of the BAT was further examined in another study using Rasch analysis, again relying on the aforementioned Flemish and Dutch representative samples. The central question was whether the four subscales of the BAT could be integrated into a single unified burnout score, and whether the items were able to differentiate between gender, age, and country (differential item functioning, DIF). The findings indicated that the BAT meets the requirements of the Rasch model, thereby confirming the existence of burnout as a latent trait. In other words, the study supports that the BAT has four subscales which can be meaningfully combined into one total burnout score. Moreover, the instrument functions equally well across men and women, younger and older participants, as well as Dutch and Flemish respondents [24].

Concerning secondary burnout symptoms, the results suggested that it is not meaningful to distinguish between psychological distress and psychosomatic complaints. The assumption is that although we can talk about different types of distress, these are so highly correlated with each other that they point to a general form of distress [7].

The internal consistency of the questionnaire exceeded .90, and the test–retest reliability (measured over a six-month interval) was also satisfactory. Convergent and discriminant validity were examined as well. Overall, both the BAT and its general version proved to be reliable and valid instruments for measuring burnout [4].

One study investigated the measurement invariance of the BAT across seven different representative national samples (the Netherlands *N* = 1500, Belgium *N* = 1500, Germany *N* = 1073, Austria *N* = 1059, Ireland *N* = 431, Finland *N* = 2299, and Japan *N* = 1032). The results showed that burnout, as measured by the BAT, was invariant across these samples, meaning that the questionnaire allows for cross-cultural comparison. In addition, across all seven samples, both the BAT and its four subscales consistently demonstrated internal consistency. This supports the conceptualization of burnout as a syndrome with four interrelated symptom clusters and confirms that the BAT is a reliable tool for its measurement [25]. A similar study conducted in four countries (Australia *N* = 200, the Netherlands *N* = 199, South Africa *N* = 197, and the United States *N* = 198) also found that BAT scores are applicable across these contexts (cross-cultural measurement invariance) and provided evidence for a two-factor model (i.e., that the items cluster into four subfactors, which can be summarized into a general burnout score) [26]. In a study involving nine countries (the Netherlands *N* = 1500, Belgium *N* = 1500, Germany *N* = 1073, Austria *N* = 1059, Ireland *N* = 431, Finland *N* = 703, Japan *N* = 1032, the Czech Republic *N* = 1020, and Norway *N* = 493), latent variable analysis using a bifactor-ESEM approach supported the validity and measurement invariance of the BAT23 total burnout score. This indicates that the BAT indeed provides a total burnout score while maintaining the meaningfulness of the four specific dimensions [6].

The shortened version of the Burnout Assessment Tool was also examined, and it was found that the short (and short general) versions are psychometrically just as sound as the original questionnaire [4]. The psychometric indicators of the short version were also confirmed in a South African sample of 660 employees, where the authors concluded that the BAT12 is a valid instrument with adequate psychometric indicators for assessing burnout risk [27].

Regarding convergent validity, BAT scores showed high correlations with other instruments used to measure burnout (e.g., Utrecht Burnout Scale, OLBI). In terms of discriminant validity, BAT scores were related to other well-being constructs such as work engagement, boredom at work, and workaholism, but remained distinguishable as a separate construct, indicating that burnout is not merely another form of workplace well-being [7]. Furthermore, BAT scores were positively associated with workplace stressors (e.g., conflicts at work), frequent absences (e.g., due to illness), and neuroticism. They were negatively associated with workplace resources (e.g., person–job fit), job satisfaction, intention to change jobs, work ability, optimism, resilience, conscientiousness, and self-efficacy [4].

Based on the studies reviewed above, as well as recent literature reviews (e.g. [28]), the BAT has been validated across multiple countries; however, its psychometric properties have not yet been examined in a Hungarian sample. Therefore, the present study examines the Hungarian workplace version of the Burnout Assessment Tool (BAT) in a Hungarian working population.

## Methods

### Sample

The study sample consisted of a total of 1,398 employed adults (66% women). Among the respondents, 28% were enrolled in higher education alongside their work. The mean age was 36.82 years (SD = 13 years, minimum = 18 years, maximum = 80 years). Most participants worked 40 h per week (73.25%) and performed their work on-site (68%); 40% were engaged in intellectual/professional occupations. In terms of the highest level of education, 30.76% of the sample had a high school diploma, and 34.23% held a university degree. The sample was diverse in terms of place of residence. Detailed demographic indicators are presented in Table 1.

**Table 1:** Demographic characteristics of the respondents

	*n*	%		*n*	%
Occupation			Highest level of education		
Student	215	15.38	Primary school (8 grades)	15	1.07
Hired worker (temps)	24	1.72	Vocational certificate (no high school diploma)	92	6.58
Employee	990	70.82	High school diploma	430	30.76
Entrepreneur	133	9.51	Technical high school (with diploma)	144	10.30
Homemaker	4	0.29	OKJ vocational training	145	10.37
Unemployed	10	0.72	Higher education vocational program	89	6.37
Retired	13	0.93	University degree or higher	483	34.55
Other	7	0.50	Missing	0	0
Missing	2	0.14	Place of residence		
Job position			Capital city	221	15.81
Manager	150	10.73	County-level city	310	22.17
White-collar worker/ Intellectual	560	40.06	Town	439	31.40
Skilled worker	177	12.66	Village	428	30.62
Semi-skilled/unskilled worker	213	15.24	Missing	0	0
Self-employed/freelancer	109	7.80	Working hours		
Other	19	1.36	Less than 20 h/week	9	0.64
Missing	170	12.16	20 h/week	118	8.44
Work setting			Between 20–40 h/week	49	3.51
On-site work	949	67.88	40 h/week or more	1024	73.25
Hybrid work	236	16.88	Flexible	11	0.79
Remote work	46	3.29	Occasional	183	13.09
Missing	167	11.95	Missing	4	0.29

### Data collection

Data collection was conducted online in the autumn of 2024 using the Microsoft Forms application and the snowball sampling method. Recruitment of participants was carried out with the involvement of university students and with the collaboration of several Hungarian universities (Eszterházy Károly Catholic University, Budapest University of Technology and Economics, and Széchenyi István University). The questionnaire was open to anyone over the age of 18 who was employed at the time of completion. Respondents were able to complete the questionnaire only after reading and accepting the informed consent statement. The first section of the questionnaire package consisted of demographic questions, followed by the psychological questionnaires.

### Measures

#### Burnout Assessment Tool

The questionnaire consists of a total of 33 items: 23 measuring primary symptoms and 10 measuring secondary symptoms, as in the original version. The subscales are structured in accordance with the original instrument; the four core dimensions are exhaustion, mental distance, impaired emotional control, and impaired cognitive control. The two secondary dimensions comprise psychological distress and psychosomatic complaints. Responses are provided on a five-point Likert scale: never, rarely, sometimes, often, and always. Scoring is based on summing the item scores within each subscale and then calculating the mean, resulting in a value between 1 and 5 for each subscale. The total score is obtained by summing all item scores of the questionnaire and calculating their mean [4].

#### The Hungarian version of the Burnout Assessment Tool

The Hungarian workplace version of the BAT was published by the original authors, and it is available on the official questionnaire website (https://burnoutassessmenttool.be/handleiding_vragenlijst_eng/). The translation process was done by Eszter Nagy and Noémi Nagy in 2019. A work and organizational professional helped with the back-translation and with the finalization of the items. After the translation, this Hungarian version was accepted by the international BAT validation team. The instrument has been tested on a Hungarian teacher sample by the translating authors, in which its psychometric indicators proved to be adequate: confirmatory factor analysis substantiated the original multidimensional factor structure as posited by the original authors [7]. The instrument demonstrated good internal and test-retest reliability as well. Furthermore, the scale’s construct validity was empirically supported by its significant convergent associations with the Maslach Burnout Inventory (MBI) subscales, aligning precisely with the theoretical framework [29]. In this research, we used their official version of the BAT, without modifying the items.

#### Basic psychological needs at work scale

The questionnaire is the workplace-adapted version of the Basic Psychological Needs Scale [30], which measures the satisfaction of basic psychological needs. The scale assesses the extent to which the three fundamental psychological needs are satisfied in the workplace environment: autonomy, competence, and relatedness. The questionnaire consists of three factors, each measured by three items, with responses given on a seven-point Likert scale. It does not contain any reverse-scored items. All three factors proved to be reliable, and test–retest reliability was also adequate. Factor analyses supported the three-factor structure of the instrument. Studies of divergent and convergent validity indicated moderate to strong positive correlations with the results of other questionnaires measuring similar constructs [31]. All of the subscales proved to be reliable in this study (Autonomy: Cronbach’s alpha = .83, McDonald’s omega = .83; Competence: Cronbach’s alpha = .69, McDonald’s omega = .74; Relatedness: Cronbach’s alpha = .81, McDonald’s omega = .82).

#### Work and meaning inventory

The original version of the Work and Meaning Inventory (WAMI) was developed by Steger and colleagues [32]. In the present study, we used the Hungarian version of the original WAMI, which consists of 10 items, including one reverse-scored item. The questionnaire has three subscales: Positive meaning of work, Meaning making through work, and Greater good motivations. It examines the perception of meaningfulness at work on a five-point Likert scale. The questionnaire was tested through confirmatory factor analysis, comparing the fit of one- and three-factor models, with the latter receiving stronger support. The instrument demonstrated consistent psychometric properties across different groups of Hungarian employees [33]. The WAMI was reliable in this research as well (Cronbach’s alpha = .90, McDonald’s omega = .91).

#### Satisfaction with life scale

The Satisfaction with Life Scale (SWLS) is designed to measure subjective well-being [34]. The modified Hungarian version of this questionnaire (SWLS-H) consists of five items and contains no reverse-scored items. It measures the items on a seven-point Likert scale, with higher scores indicating greater life satisfaction. The internal consistency of the scale was found to be very good, and its structural validity was excellent. Moderate positive correlations were observed with other positive psychology measures [35]. The indices indicated good reliability in present research: Cronbach’s alpha = .88, McDonald’s omega = .87.

#### Satisfaction with work scale

This questionnaire measures work-related satisfaction. It was developed by adapting the Satisfaction with Life Scale (SWLS), with items reformulated to focus on work. The questionnaire also consists of five items, with responses provided on a seven-point Likert scale. In previous research, the scale demonstrated excellent internal reliability [36], and another study examining factorial validity found a good model fit [33]. The reliability was good in this research (Cronbach’s alpha = .87, McDonald’s omega = .88).

### Data analysis

Data were analyzed using JASP statistical software, version 0.19.3 [37]. Confirmatory factor analysis (CFA) was conducted using the maximum likelihood method, with robust (sandwich) standard error estimation. To evaluate the final model fit, the following indices were considered: the χ² statistic (with χ²/df), which traditionally indicates good fit at a *p*-value of .05, however, with large samples, it is almost always significant [38]; the standardized root mean square residual (SRMR) index, where values below 0.08 indicate acceptable fit [39]; the root mean square error of approximation (RMSEA), where values between 0.10 and 0.08 indicate mediocre fit and values below 0.08 indicate good fit [40]; the confirmatory factor index (CFI) and Tucker–Lewis index (TLI), with values above 0.90 indicating good fit [39]. To test group invariance by sex (male vs. female), work setting (office-based, hybrid, or remote), and higher education student status (yes vs. no), multiple-group factor analysis (MG-CFA) was performed. During the analysis, a series of increasingly constrained CFA models were run, and following international literature [41], invariance was determined based on differences in CFI values, with differences below 0.01 indicating invariance. Reliability was assessed using Cronbach’s alpha and McDonald’s omega, with a threshold of .7 [42, 43]. Average interitem correlations were also considered, with acceptable values ranging from .30 to .90 [44]. To test construct validity, AVE (average variance extracted) values were used with the threshold of ≥ 0.05 [45]. We also calculated HTMT (heterotrait-monotrait ratio) to assess discriminant validity with the threshold of < 0.85 [46].

## Results

### Confirmatory factor analysis, multiple-group confirmatory factor analysis, and reliability testing

During the CFA, two models were tested. A model, without second-order factors, and a second-order model proposed by the original authors [7].

**Fig. 1:**
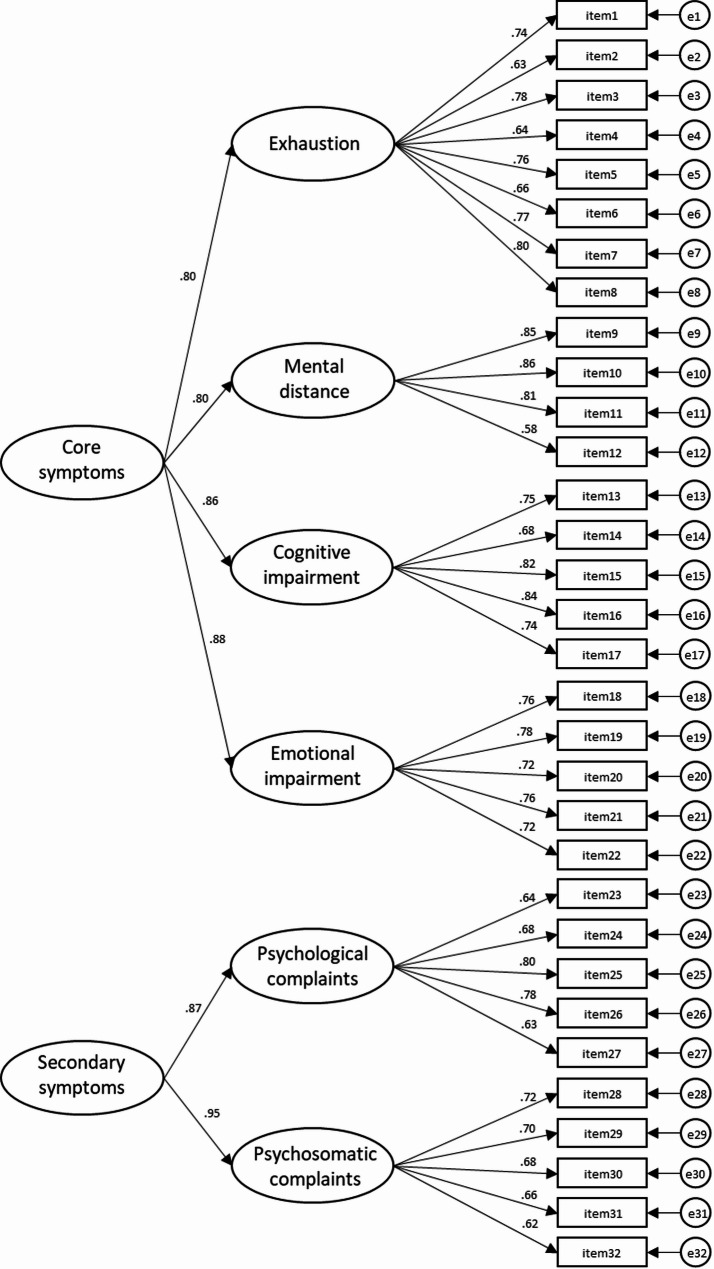
Factor structure of the BAT. Note: Standardized factor loadings are presented in the figure

The first model fit indices were as follows: χ² = 2904.62, χ² /df = 6.47, *p* < .001; SRMR = 0.05; RMSEA = 0.06 (CI = 0.06–0.07), CFI = 0.90; TLI = 0.89. Although the fit indices were mostly acceptable, the factor variances were quite high (between 0.83 − 050). We tested the second-order model, the fit indices indicated also an adequate fit (χ² = 2084.55, χ² /df = 4.58, *p* < .001; SRMR = 0.05; RMSEA = 0.06 (CI = 0.06–0.07), CFI = 0.90; TLI = 0.89). The variances between the second-order factor was high as well (the standardized estimate was 0.79). Factor loadings ranged between 0.58 and 0.95. While the fit indices were mostly identical, because of theoretical considerations, we decided on the latter, second-order model. The final model, including standardized factor loadings and relationships among alpha factors, is presented in Fig. 1.

The multiple-group factor analysis (MG-CFA) was conducted for three groups. Measurement invariance could be assumed across gender (male vs. female), work setting (office work, hybrid work, and remote work; see Table 2). Regarding university student status, it is important to note that the CFI values were at the threshold, meaning that the scalar invariance cannot be unequivocally assumed.

**Table 2:** Results of the MG-CFA analysis

	CFI	Δ CFI
Gender		
Configural	0.890	
Metric	0.889	0.001
Scalar	0.890	0.001
Work form		
Configural	0.870	
Metric	0.870	0
Scalar	0.870	0
University student status		
Configural	0.880	
Metric	0.880	0
Scalar	0.890	0.010

Reliability was adequate according to the reliability indices for the subfactors, main factors, and the total questionnaire score. The average inter-item correlations were also satisfactory (see Table 3).

**Table 3:** Reliability indicators and mean scores

	Coefficient α	Coefficient ω	Average interitem correlation	Mean scores
Core symptoms	.94	.94	.43	2.27
Exhaustion	.90	.90	.52	2.69
Mental distance	.85	.86	.59	2.18
Cognitive impairment	.87	.87	.58	2.04
Emotional impairment	.86	.86	.56	1.91
Secondary symptoms	.88	.88	.43	2.26
Psychological complaints	.83	.83	.50	2.49
Psychosomatic complaints	.81	.81	.46	2.03
Total score of burnout	.95	.95	.38	2.27

### Validity testing

To test the convergent validity of the BAT, we calculated AVE values. The AVE indicated appropriate validity: factors measuring core symptoms were above the threshold (> 0.05), but the factors related to the secondary symptoms were at the threshold: Exhaustion = 0.53, Mental distance = 0.61, Cognitive impairment = 0.60, Emotional impairment = 0.60, Psychological complaints = 0.50, Psychosomatic complaints = 0.50).

We also conducted correlation analysis with constructs that in previous research have shown associations with burnout. The BAT total score indicating burnout demonstrated a negative, medium-sized relationship with life and work satisfaction, the satisfaction of basic psychological needs at work (autonomy, competence, and relatedness), and the meaningfulness of work. The BAT subscales showed similar patterns of associations (see Table 4). However, in the case of several correlations, the covariances between subfactors had quite low effect sizes. This was most evident in the case of factors measuring secondary symptoms (psychological and psychosomatic complaints) and between competence, relatedness, and meaningful work.

**Table 4:** Correlations between burnout subscales and work-related variables

	SWWS	SWLS	Autonomy	Competence	Relatedness	WAMI
Exhaustion	− .39^***^	− .25^***^	− .36^***^	− .32^***^	− .29^***^	− .26^***^
Mental distance	− .55^***^	− .32^***^	− .49^***^	− .49^***^	− .40^***^	− .53^***^
Cognitive impairment	− .28^***^	− .24^***^	− .29^***^	− .30^***^	− .24^***^	− .26^***^
Emotional impairment	− .31^***^	− .26^***^	− .30^***^	− .30^***^	− .28^***^	− .28^***^
Psychological complaints	− .30^***^	− .28^***^	− .26^***^	− .24^***^	− .19^***^	− .19^***^
Psychosomatic complaints	− .23^***^	− .22^***^	− .22^***^	− .19^***^	− .15^***^	− .17^***^
Total score of burnout	− .44^***^	− .33^***^	− .40^***^	− .38^***^	− .32^***^	− .35^***^

To measure discriminant validity, we used HTMT values. All of the values were acceptable (HTMT values ranged between 0.52 − 0.83)

*SWWS* Satisfaction With Work Scale, *SWLS* Satisfaction With Life Scale, *WAMI* Work And Meaning Inventory

^*^p < .01, ^***^p < .001

### Descriptive statistics and subgroups differences

Regarding gender differences, although a significant result was found in the total burnout score in favor of women, the effect size was negligible. No significant differences were observed in the Primary symptoms factor or its corresponding subfactors. In the Secondary symptoms factor, women scored significantly higher with a small effect size, and this difference in favor of women was observed across both subfactors (see Table 5).

**Table 5:** Gender differences analysis

	Gender	*n*	Mean	SD	Mann-Whitney test of difference
Exhaustion	Man	474	2.66	0.80	U = 211565.5, *p* = .31, *r* = − .03
	Woman	923	2.70	0.81	
Mental distance	Man	474	2.23	0.93	U = 231084.5, *p* = .08, *r* = .06
	Woman	922	2.15	0.94	
Cognitive impairment	Man	474	2.05	0.79	U = 221899.5, *p* = .63, *r* = .02
	Woman	922	2.03	0.79	
Emotional impairment	Man	473	1.91	0.73	U = 222833.5, *p* = .52, *r* = .02
	Woman	923	1.90	0.78	
Psychological complaints	Man	473	2.30	0.83	U = 179625.5, *p* < .001, *r* = − .18
	Woman	923	2.59	0.93	
Psychosomatic complaints	Man	474	1.79	0.73	U = 159,774, *p* < .001, *r* = − .27
	Woman	923	2.15	0.83	
Core symptoms	Man	473	2.27	0.67	U = 219,333, *p* = .83, *r* < .001
	Woman	921	2.27	0.70	
Secondary symptoms	Man	473	2.04	0.70	U = 166,460, *p* < .001, r = .-24
	Woman	923	2.37	0.81	
Total score of burnout	Man	472	2.20	0.63	U = 199,039, *p* < .001, r = .-08
	Woman	921	2.30	0.68	

Age-based differences in burnout were also examined. The age groups were defined as follows: (1) 18–30 years (2), 31–45 years (3), 46–55 years, and (4) 56 years and older. Significant differences were found between the groups (*F*(3,423.56) = 19.22, *p* < .001, η² = 0.04). Post hoc analyses revealed significant differences between groups (1) and (2) (*t* = -5.70, *p*_*dunnett*_ < .001) (1) and (3), (*t* = -6.05, *p*_*dunnett*_ < .001), and (1) and (4) (*t* = -4.92, *p*_*dunnett*_ < .001), indicating that early-career individuals reported the highest burnout levels. The descriptive statistics table (Table 6) further shows a trend of decreasing burnout scores with increasing age

**Table 6:** Burnout scores by age and job position

Age	*n*	Mean	SD
18–30 years	540	2.43	0.69
31–45 years	417	2.19	0.66
46–55 years	337	2.15	0.61
over 56 years	100	2.08	0.55

Job position	*n*	Mean	SD
Manager	149	2.04	0.56
Intellectual/white-collar worker	558	2.26	0.63
Skilled worker	177	2.34	0.66
Semi-skilled/unskilled worker	212	2.50	0.63
Self-employed/freelancer	109	2.13	0.65

A significant difference was found based on university student status, with students who are also employed showing significantly higher burnout. This result applies to the primary symptoms of burnout: the student group reported higher exhaustion and impaired cognitive control with a very small effect size, while differences in mental distance and impaired emotional control were not significant. For secondary symptoms, students also showed higher scores, but the effect sizes were quite small here as well, with a significant difference observed for psychological complaints but not for psychosomatic complaints. For detailed results, see Table 7

**Table 7:** Differences by university student status

	University student status	*n*	Mean	SD	Mann-Whitney test of difference
Exhaustion	No	831	2.65	0.79	U = 150746.5, *p* < .001, *r* = − .10
	Yes	401	2.78	0.79	
Mental distance	No	831	2.18	0.91	U = 156,999, *p* = .11, *r* = − .06
	Yes	400	2.28	0.95	
Cognitive impairment	No	830	1.99	0.73	U = 148,710, *p* < .001, *r* = − .11
	Yes	401	2.16	0.84	
Emotional impairment	No	831	1.88	0.69	U = 158572.5, *p* = .19, *r* = − .05
	Yes	400	1.98	0.82	
Psychological complaints	No	831	2.41	0.85	U = 137724.5, *p* < .001, *r* = − .17
	Yes	400	2.69	0.94	
Psychosomatic complaints	No	831	1.99	0.76	U = 162,662, *p* = .5, *r* = − .02
	Yes	401	2.05	0.84	
Core symptoms	No	830	2.24	0.65	U = 149089.5, *p* < .001, *r* = − .10
	Yes	399	2.36	0.71	
Secondary symptoms	No	831	2.2	0.74	U = 146505.5, *p* < .001, *r* = − .12
	Yes	400	2.37	0.81	
Total score of burnout	No	830	2.23	0.62	U = 145444.5, *p* < .001, r = .-12
	Yes	398	2.36	0.67	

Regarding job position, the groups (managers, white-collar workers/intellectuals, skilled workers, semi-skilled workers, self-employed/entrepreneurs) showed significant differences in burnout scores (*F*(5,159.74) = 11.91, *p* < .001, *η²* = 0.04). Post hoc analyses indicated that managers had significantly lower burnout scores compared to intellectual/white-collar workers (*t* = 3.78, *p*_*dunnett*_ < .001), skilled workers (*t* = 4.28, *p*_*dunnett*_ < .001), and unskilled workers (*t* = 6.84, *p*_*dunnett*_ < .001). No significant differences were found between managers and entrepreneurs, nor between skilled and semi-skilled workers. Regarding work setting (office-based, hybrid, remote), no significant differences in burnout were observed (*F*(2, 1224) = 1.14, *p* = .32, *η²* < 0.001).

## Discussion

The present study examined the psychometric properties of the Burnout Assessment Tool (BAT) in a Hungarian sample. Our results indicate that the BAT in the Hungarian sample follows the structure defined by the original authors [7], and that invariance can be assumed across gender, university student status, and work setting. That is, the factor structure, factor loadings, and means can be considered equivalent across these groups.

The Hungarian version of the BAT demonstrated satisfactory psychometric properties, confirming the model proposed by the original authors and showing adequate reliability. Within the BAT model, impaired cognitive and emotional control is conceptualized as a core component of burnout; however, its theoretical role has received less attention. Our results suggest that this dimension may reflect difficulties in self-regulation that emerge under prolonged need frustration, thereby linking symptom-level manifestations of burnout to underlying motivational processes. The negative relationship between the overall burnout score and the satisfaction of basic psychological needs at work highlights the importance of continuously monitoring and supporting the fulfillment of autonomy, competence, and relatedness needs in workplace or educational settings, as this may be associated with lower levels of burnout and may have implications for prevention. The strong associations observed for mental distance are also theoretically meaningful. Within the BAT framework, mental distance reflects psychological withdrawal from work [7], while from a self-determination theory perspective, it may represent a response to the chronic frustration of basic psychological needs for autonomy, competence, and relatedness [47]. When these needs are not sufficiently satisfied, individuals may become less engaged and increasingly detached from their work, which is consistent with previous findings showing that need satisfaction is positively associated with well-being and engagement, and negatively associated with distress and burnout [48].

In line with this interpretation, burnout and engagement can be understood as opposing yet related indicators of occupational well-being [49]. Whereas engagement is characterized by vigor and dedication, burnout involves exhaustion and withdrawal. Our findings support the notion that mental distance may represent an important process linking reduced need satisfaction to diminished engagement and increased burnout symptoms. Paying attention to these factors in preventive efforts may be associated with lower levels of disengagement from work and social withdrawal.

We found similar correlational results between burnout and life satisfaction, job satisfaction, and the experience of meaningful work. It is also important to pay attention to the role of engagement, identified as the “antipode” of burnout [50], since engagement represents a productive and fulfilling state. Among the variables mentioned, the meaningfulness of work plays a particularly important mediating role. Individuals who perceive their work as meaningful show greater engagement with their job [32, 51, 52], and meaningful work has a positive impact not only on general well-being [53] but also on life satisfaction and the experience of meaning in life [54, 55]. Experiencing meaningfulness may be considered a psychological resource that is associated with both physical and psychological well-being and lower levels of perceived strain in stressful situations [56].

When examining group differences, no significant differences were found between men and women in primary symptoms and their subfactors. However, women scored significantly higher on secondary symptoms and their subfactors. These results (e.g., women’s higher emotional exhaustion) are consistent with international literature [57–60] and highlight the influence of gender-specific coping strategies and expectations. Regarding age differences, our findings suggest that early-career individuals are the most at risk for burnout, while the likelihood of burnout decreases later in life.

The level of burnout differed not only by age and gender, but also by university student status. However, due to the low effect sizes, these differences cannot be interpreted as significant group differences. There could be key confounding variables (e.g., age, education, working hours) that were not controlled in the analysis. Observed differences may reflect demographic composition rather than true occupational effects.

The analysis of age groups confirmed higher burnout among early-career individuals, with burnout scores decreasing progressively with age. No significant differences in burnout were found with respect to work setting.

Based on the comparison of different occupational groups, white-collar employees, skilled workers, and semi-skilled workers reported higher levels of burnout. This finding aligns with the established notion in the literature that workload, level of autonomy, and the work environment play a significant role in the development of burnout [50]. Previous research [61] has also identified lower quality of life and higher workplace stress among blue-collar workers, which may be explained by low autonomy combined with both physically and mentally demanding tasks. These results underscore the need to develop targeted burnout prevention programs for these groups and to continuously examine the underlying causes. Preventive interventions can contribute to employee well-being, the maintenance of workplace performance, and reduced turnover.

Psychosomatic symptoms were not consistently associated with high burnout levels, suggesting that exhaustion and experienced psychological distress do not always manifest in physical symptoms—particularly among young adults, who may be less likely to recognize or express signs of mental and physical overload.

Our findings indicate that the BAT is a valid and reliable instrument for measuring the complex phenomenon of burnout in Hungarian samples as well. Beyond its theoretical relevance, our results also point to concrete avenues for intervention in both educational and workplace contexts.

### Limitations of the study and future directions

The present study has several limitations that should be acknowledged. First, the use of non-probability sampling limits the generalizability of the findings, as the sample is not fully representative of the Hungarian workforce across demographic and occupational groups. Future studies should therefore aim to replicate these findings in more representative samples and within specific occupational cohorts.

Second, although the goodness-of-fit indices supported the proposed factor structure, they did not indicate a perfect model fit, suggesting that additional latent complexities may remain. Furthermore, measurement invariance testing indicated that, in the subgroup of university students, CFI values were at the threshold of acceptability. Accordingly, full invariance cannot be unequivocally assumed for this group, and comparisons involving student populations should be interpreted with caution. Third, the analyses of demographic differences were primarily descriptive and did not account for potential confounding variables (e.g., age, education, working hours), which may underline the observed patterns. Future research should employ more complex analytical approaches to better understand these relationships.

Finally, for subsequent research involving more sophisticated structural models (e.g., structural equation modeling or latent profile analysis), it is advisable to employ more specialized statistical software such as Mplus or R. These platforms offer more control over the analyses.

In addition, the cross-sectional design of the study does not allow for causal interpretations, and the exclusive reliance on self-report measures may introduce common method bias. Future research should address these limitations by applying longitudinal designs and multi-method approaches. In particular, the assessment of test–retest reliability and predictive validity would further strengthen the psychometric evaluation of the Hungarian BAT.

Finally, future studies may benefit from applying more advanced statistical techniques (e.g., structural equation modeling or latent profile analysis) using specialized software (e.g., R or Mplus) to further explore the underlying structure of burnout.

Overall, the present findings provide a foundation for future research in the Hungarian context and contribute to cross-cultural investigations of burnout. Further studies are needed to examine the applicability of the BAT across diverse occupational groups, particularly in high-stress professions, where the manifestation of burnout may differ.

## Conclusions

The Burnout Assessment Tool (BAT) was developed to address conceptual and psychometric limitations of earlier instruments, such as the Maslach Burnout Inventory [2]. It is based on a multidimensional conceptualization of burnout, encompassing core symptoms (exhaustion, mental distance, and impaired emotional and cognitive control) and secondary symptoms (psychological distress, psychosomatic complaints and depressive mood). The present study examined the psychometric properties of the Hungarian workplace version of the BAT in a large sample. The findings support the original factor structure and indicate that the instrument demonstrates adequate reliability and validity in the Hungarian context.

However, conceptual, psychometric, and technical issues have been identified with this tool [7]. For this reason, the Burnout Assessment Tool (BAT) was developed, which, as a first step in the process, was accompanied by the formulation of a new conceptualization of burnout. The core symptoms of burnout identified within this framework include exhaustion, mental distance, and impaired emotional and cognitive control, while the secondary symptoms encompass psychological distress, psychosomatic complaints, and depressive mood [4]. The tool can be applied to both individual and group assessments of burnout. Studies conducted in different countries have confirmed that both the full and the short versions of the questionnaire are valid and reliable instruments with adequate psychological properties for measuring burnout. In the present study, the BAT was examined on a Hungarian sample, involving a total of 1,398 participants. Overall, the Hungarian version of the BAT can be considered a reliable and valid tool for assessing burnout, contributing to both national and cross-cultural research and offering a useful instrument for future research and applied settings.

## Data Availability

The datasets used and/or analysed during the current study are available from the corresponding author on reasonable request.
